# The Inter-National Hahnemannian Association

**Published:** 1881-06

**Authors:** 


### HOMŒOPATHIC PHYSICIAN.

A MONTHLY JOURNAL OF MEDICAL SCIENCE.

"If our school ever gives up the strict inductive method of Hahnemann, we are lost, and deserve to be mentioned only as a caricature, in the history of medicine." — CONSTANTINE HERING.

Vol. I. JUNE, 1881. No. 6.

### EDITORIAL.

THE INTERNATIONAL HAHNEMANNIAN ASSOCIATION.—"On the 10th of April, 1844, there met a convention of the practitioners of homœopathy in the United States, at the Lyceum of Natural History, in the City of New York.

"A preamble and resolutions, in these words, were adopted: Whereas, a majority of the allopathic physicians continue to deride and oppose the contributions to the Materia Medica that have been made by the homœopathic school; and whereas, the state of the Materia Medica in both schools is such as imperatively to demand a more satisfactory arrangement and greater purity of observation, which can only be obtained by associate action on the part of those who diligently *seek for truth alone*;* and, inasmuch as the state of public information respecting the principles and practice of homœopathy is so defective as to make it easy for *mere pretenders*, to this very difficult branch

* Italics ours.—ED.

of the healing art, to acquire credit as proficients in the same; Therefore Resolved: That it is deemed expedient to establish a society entitled "The American Institute of Homœopathy;" and the following are declared to be the essential purposes of said Institute:

1st. The reformation and augmentation of the Materia Medica.

2d. The *restraining of physicians from pretending to be competent to practice homœopathy who have not studied it in a careful and skilful manner.*"*

Such was the organization and such were the purposes of the American Institute of Homœopathy. First, to enlarge and purify the Materia Medica, and secondly, to see that none practiced homœopathy who had not "studied it in a careful and skilful manner." Two noble purposes; how have they been carried out? Has the American Institute of Homœopathy always striven to accomplish these "essential purposes of its organization"? Are its sessions and work devoted to the "reformation and augmentation of the Materia Medica"? Are its members those who have "studied homœopathy in a careful and skilful manner"? Another purpose of the founders of this Institute, was evidently to enlighten the laity as to the principles of true homœopathy, for they complained that "the state of *public information respecting the principles and practice of homœopathy is so defective as to make it easy for mere pretenders, to this very difficult branch of the healing art to acquire credit as proficients in the same.*"* Has the American Institute ever attempted to so enlighten the laity that they could discriminate between the homœopath and the "mere pretender"?* To one and all of these questions there can be but one honest answer—a sorrowful no!

---

* That many "mere pretenders" still exist, Dr. Dunham testified in these words:

"There are among those who call themselves homœopathists, some who are impostors; men unworthy to be called physicians; men without knowledge and without conscience, who play upon the credulity of mankind, and pervert to their own aggrandizement every trust committed to them. That such men, professing to be of our school, should be regarded by the community as belonging to it, and should tarnish our fair name by their foul deeds, is certainly a misfortune."—*N. A. J. of Homoeopathy*, vol. xix, p. 144.

(1). As the Materia Medica is our chief, and in fact our only means for curing disease, it behooves us *first* to perfect it, and *then* to study the minor branches of medicine. (2). As it is very necessary to the growth and reputation of homœopathy that none be recognized as homœopathic physicians who have not "studied homœopathy in a careful and skilful manner." (3). As these two cardinal purposes of its organization have been purposely and perpetually neglected by the American Institute of Homœopathy. (4). As those who desired to "seek diligently for truth alone" found themselves outnumbered by the eclectic, mongrel, or mixed element—what ever you please to call it—of the Institute. Therefore, in view of these four facts, the seekers "for truth alone" were compelled to organize a medical society where "associate action on the part of those who diligently seek for truth alone" would be effective. Hence the *International Hahnemannian Association*. As to the purposes of this Association we read:

"Whereas, we believe the "Organon of the Healing Art," as promulgated by Samuel Hahnemann to be the only reliable guide in therapeutics, and

"Whereas, this [guide] clearly teaches that homœopathy consists in the law of the similars, the totality of the symptoms, the single remedy, the minimum dose of the dynamized drug, and these not singly but collectively; and

"Whereas, numbers of professed homœopathists not only violate these tenets, but largely repudiate them; and

"Whereas, an effort has been made on the part of such physicians to unite the homœopathic with the allopathic school; therefore

"Resolved, that the time has fully come when legitimate Hahnemannian homœopathists should publicly disavow all such innovations;

"Resolved, that the mixing or alternating of two or more medicines is non-homœopathic;

"Resolved, that in non-surgical cases we disapprove of medicated topical applications and mechanical appliances as being also non-homœopathic;

"Resolved, that as 'the best dose of medicine is ever the smallest,' we can not recognize as being homœopathic such treatment as suppresses symptoms by the toxic action of the drug;

"Resolved, that we have no sympathy in common with those physicians who would engraft on homœopathy the crude ideas and doses of allopathy or eclecticism, and we do not hold ourselves responsible for their 'fatal errors' in theory, and failures in practice;

"Resolved, that as some self-styled homœopathists have taken occasion to traduce Hahnemann as a 'fanatic,' as 'dishonest,' and as 'visionary,' and his teaching as 'not being the standard of homœopathy of to-day,' that we regard all such as recreant to the best interests of homœopathy;

"Resolved, that for the purpose of promoting these sentiments, and for our mutual improvement, we organize ourselves into an *International Hahnemannian Association*, and adopt the following constitution and by-laws."* The constitution, moreover declares that no one can be elected a member of the Association until he sign the declaration of principles, governing that body.

Thus we see that the founders of the International Hahnemannian Association have in view substantially the same purposes as had the originators of the American Institute of Homœopathy; namely, to improve our therapeutics and restrain unfit and unprincipled persons from practicing homœopathy.

If it was right and necessary for the originators of the American Institute of Homœopathy to declare in 1844 that our Materia Medica needed augmentation and improvement, and that many unfit persons were practicing homœopathy, it was certainly equally right and much more necessary for the founders of the International Hahnemannian Association to make the *same* declaration in 1880. Had the American Institute of Homœopa-

*The Organon*, vol. iii, pp. 454-5.

thy done its full duty during the thirty-six years intervening between these organizations, the formation of this new and International Association would have been unnecessary.

The perversions, which the (so called) rational and progressive homœopaths are seeking to make in our therapeutics, are too many and too injurious to need a recapitulation at this time.

That many are practicing something which they call homœopathy, needs no demonstration; the journals teem with their effusions; the society debates and the transactions are full of this rubbish. There may be some wheat amid all these tares but it would take a life's work to winnow it out. The founders of the International Hahnemannian Association desire to have the wheat without the tares, so they propose to gather *their* golden grain into a separate store house, and allow those who will amuse themselves with the tares.

The declaration of principles put forth by this Association is substantially the same as that declaration which was signed by many, some years ago, and whose signers are now known as "The Legion of Honor." All those who joined the "Legion of Honor" because they were desirous of seeing its principles triumph, should join this Association and *work* for those principles.

These principles are briefly those that the HOMŒOPATHIC PHYSICIAN put forth as its "platform:"

The Law of the Similars.
The Single Remedy.
The Minimum Dose.

The law of the similars teaches that a drug must be proved on the healthy before being used on the sick: the single remedy teaches that alternation and combination are non-homœopathic: the minimum dose teaches that it is unwise to give a larger dose than is necessary to cure, for a too large dose of the simillimum remedy *will* produce an undesired aggravation. This minimum dose may in one case be the 30th, while in another it may be the C.M. attenuation. The single remedy and the minimum dose both forbid topical (medicinal) applications.

It has been charged by some that the International Hahnemannian Association (and also this journal) advocate high potencies exclusively. This is not so. While the experience of its members undoubtedly points to the greater efficacy of the high potencies, its principles advocate any dose necessitated by a strict following out of the law of the similars. Again it has been charged that the Association favored "Isopathy." This is also false. But a word as to isopathy. Any remedy—be it aconite or syphilinum—which has been proved on the healthy, and has produced legitimate and characteristic symptoms may be prescribed according to our law, and is then *not isopathic but homœopathic.* Indeed, there is no such thing as an "isopathic symptom." "Isopathy" simply means to give the product of a disease for that disease without regard to *symptoms;* it is in fact seeking a specific for a pathological disease. This is empiricism not homœopathy. The International Hahnemannian Association repudiates all empiricism, whether it comes in the form of the crude drug or in the C.M. potency, aconite or syphilinum.

Thus, we have endeavored to briefly outline the causes which led to the organization of this Association, and the essential features of its work. We have seen that these causes are similar to those which led to the organization of the American Institute of Homœopathy thirty-six years previous. The Institute, having failed to execute "the essential purposes" of its organization, the International Hahnemannian Association proposes to execute its neglected work, and thereby regenerate the homœopathic school.

Of this Association Dr. Smythe—an allopath—wrote: "Carroll Dunham's address was only the exciting cause of the schism which took place in the ranks of homœopathy. It had been gathering form for a long time, and must have come sooner or later; in fact, it could not have been delayed much longer. There are now two wings to the school, the liberals and the straight-jackets. A house can not stand which is divided against itself. The liberals will necessarily become eclectics and the straight-jackets will return to Hahnemannism, pure and unadulterated. Preliminary steps to accomplish this end have already been taken. During the meeting of the American Institute of Homœopathy, at Milwaukee, June, 1880, *The International Hahnemannian Association* was formed..... The formation of this Association, and the adoption of its platform is a return to the pure, inflexible, dogmatic homœopathy of Hahnemann."

The work which the International Hahnemannian Association has undertaken, is a noble one, and a great one. It is to stem the torrent of eclecticism which threatens to wash away all the old, safe, reliable landmarks left us by Hahnemann. This work necessitates the increase of the Materia Medica, and its purification from all errors—clinical, pathological, or hypothetical; the regeneration of its medical schools; the instruction of its physicians and the exposure of false theory and erroneous opinion of many of its professors and leaders. This task the International Hahnemannian Association proposes to accomplish by a strict adherence to the law and its corollaries and by a full and clear explanation and illustration of them. The members of the International Association retain their membership in the Institute, hoping — almost against hope — that a little leaven may eventually leaven the whole. To accomplish this the better the two societies will meet at same time and place.

Now, let all true homœopaths, all those who believe, who *know* from experience, that the law of the similars, with these corollaries, is the best, the safest, and in fact the only true guide in healing the sick; let all such join this Association and save that law from being swallowed up in empiricism.

* See, "Medical Heresies," pp. 215–7.

